# MicroRNA regulatory networks in the pathogenesis of sarcopenia

**DOI:** 10.1111/jcmm.15197

**Published:** 2020-04-12

**Authors:** Jiayu Yin, Zhiyuan Qian, Yuqi Chen, Yi Li, Xiang Zhou

**Affiliations:** ^1^ Department of Cardiology The Second Affiliated Hospital of Soochow University Suzhou China; ^2^ Department of Neurosurgery The Second Affiliated Hospital of Soochow University Suzhou China

**Keywords:** microRNAs, pathogenesis, Sarcopenia, treatment

## Abstract

Sarcopenia is an age‐related disease characterized by disturbed homeostasis of skeletal muscle, leading to a decline in muscle mass and function. Loss of muscle mass and strength leads to falls and fracture, and is often accompanied by other geriatric diseases, including osteoporosis, frailty and cachexia, resulting in a general decrease in quality of life and an increase in mortality. Although the underlying mechanisms of sarcopenia are still not completely understood, there has been a marked improvement in the understanding of the pathophysiological changes leading to sarcopenia in recent years. The role of microRNAs (miRNAs), especially, has been clearer in skeletal muscle development and homeostasis. miRNAs form part of a gene regulatory network and have numerous activities in many biological processes. Intervention based on miRNAs may develop into an innovative treatment strategy to conquer sarcopenia. This review is divided into three sections: firstly, the latest understanding of the pathogenesis of sarcopenia is summarized; secondly, increasing evidence for the involvement of miRNAs in the regulation of muscle quantity or quality and muscle homeostasis is highlighted; and thirdly, the possibilities and limitations of miRNAs as a treatment for sarcopenia are explored.

## 1 INTRODUCTION

The escalating proportion of elderly individuals in developed societies has led to a year‐on‐year increase in geriatric diseases. Improving quality of life for the elderly has thus become a very real problem that society must face. The term “sarcopenia” was first proposed to describe age‐related loss of muscle mass and strength by Irwin Rosenberg in 1989. Initially, sarcopenia was generally considered to be a complication of ageing,^1^ but was officially included in the ICD‐10‐MC Diagnosis Code in 2016, marking it as an independent disease, which has since attracted widespread attention worldwide.^2^ The effects of ethnicity, environment, age and gender on quantity and quality of skeletal muscle have been investigated, and the definition and diagnosis of sarcopenia are being constantly updated. In 2018, the second European Working Group on Sarcopenia in Older People (EWGSOP2) provided an updated definition of sarcopenia as an age‐related disease with a decline in skeletal muscle quantity or quality and a decrease in skeletal muscle strength or physical performance.^3^ The extent of muscle atrophy in 65‐year‐olds is 5‐15% and as high as 50% in people over 80 years old, the majority of whom suffer from complications or comorbidities, including falls, fractures, osteoporosis, frailty, and cachexia, leading to increased mortality.^4^

MicroRNAs (miRNAs) are small, ubiquitous and non‐coding RNA molecules, containing approximately 22 nucleotides, which are highly conserved in eukaryotes. By base‐pairing with complementary sequences within mRNA molecules, miRNAs silence mRNA by inhibiting translation or allowing degradation of mRNA. miRNAs thus act as powerful regulators of gene expression at the post‐transcriptional level. Each individual miRNA is capable of targeting diverse mRNAs and, additionally, each individual mRNA can be modulated by various miRNAs.^5^ Large numbers of miRNAs have been identified over recent years by comprehensive analyses of mammalian transcripts. miRNAs act as part of a gene regulatory network and control numerous biological processes, as well as showing abnormal expression in many disease states.^6^ The in‐depth studies of miRNAs are of great importance for the diagnosis, treatment and prognosis of diseases, and recently, miRNAs have been confirmed to be closely associated with the occurrence and development of sarcopenia.^7^

Sarcopenia is an important manifestation of ageing and causes a gradual decline in physiological functions in the elderly, leading to decreased quality of life and even reduced life expectancy. Because of confused definitions and inaccurate screening tools, sarcopenia remains undiagnosed in most cases. Crucially, other than increasing exercise and improving nutrition, which may provide some benefit, there is no safe and effective intervention for sarcopenia. Effective biomarkers and treatments are thus urgently needed to overcome sarcopenia. With the development of high‐throughput sequencing technology, a large number of miRNA molecules, which differ in sequence, structure, expression and function, have now been associated with sarcopenia. This review will summarize the latest understanding of the pathophysiological changes that take place in sarcopenia and highlight the increasing evidence associating miRNAs with sarcopenia (Table 1).

**TABLE 1:** Summary of miRNA regulatory networks in sarcopenia

Pathophysiology	miRNAs	Targets	References
Satellite cells	miR‐1	Pax7	59, 60
	miR‐206	Pax7	59, 60
	miR‐486	Pax7	59, 60
	miR‐27	Pax3, Mef2C	62, 63
	miR‐31	Myf5	^64^
	miR‐195	*Cdc25, Cnd*	^65^
	miR‐497	*Cdc25, Cnd*	^65^
	miR‐489	*DEK*	^76^
	Let‐7b/e	Pax7	^67^
Protein homeostasis	miR‐199‐3p	IGF‐1, mTOR, RPS6K	^70^
	miR‐125b	IGF‐2	^68^
	miR‐223	IGF‐2	^69^
	miR‐133	IGF‐1R	^71^
	miR‐195	IR, IGF‐1R	^72^
	miR‐497	IR, IGF‐1R	^72^
	miR‐487b‐3p	IRS1	^73^
	miR‐128	IR, IRS1, PIK3R1	74, 75
	miR‐106a‐5p	PI3K	^76^
	miR‐432	P55PIK	^77^
	miR‐29	Akt3	^79^
	miR‐21	TGF‐βI	^78^
	miR‐26a	Smad1	^80^
	miR‐27	myostatin, Smad3	82, 83
	miR‐128	myostatin	^75^
	miR‐22	TGF‐βR1	^81^
	miR‐675‐3p	Smad1, Smad5	^84^
	miR‐24‐3p	Smad2	^85^
	miR‐23a	MuRF1, MAFbx	^86^
	miR‐27a	MuRF1, MAFbx	^86^
	miR‐351	MuRF1, MAFbx	^87^
	miR‐29c	MuRF1	^88^
	miR‐486	FoxO1, PTEN	^89^
Muscle fibre types	miR‐208b	Sp3, Sox6, HP‐1β, Purβ	^91^
	miR‐499	Sp3, Sox6, HP‐1β, Purβ	^91^
Mitochondria and ROS	miR‐1	mtCox1, mtNd1, mtCytb, mtCox, mtAtp8	^95^
	miR‐696	PGC‐1α	^96^
	miR‐23	PGC‐1α,	^98^
	miR‐34a	Bcl‐2	^99^
	miR‐146a	Bcl‐2	^99^
	miR‐181a	Bcl‐2	^99^
	miR‑340‑5p	Nrf2	^11^
	miR‐206	Nrf2	^12^
Neurodegeneration	miR‐206	HDAC4	^13^
	miR‐375	P53	^15^
	miR‐146a	NOTCH	^16^
	miR‐234	ACH	^18^
	MiR‐1	ACH	^111^
	let‐7	*abrupt*	109, 110
	miR‐125	*abrupt*	109, 110
Fat infiltration	miR‐1‐2	BAF subunits	^114^
	miR‐133	BAF subunits, PRDM16	114, 120
	miR‐206	BAF subunits	^114^
	miR‐499	PRDM16	^116^
	miR‐23a	PDGFRα	117, 118
	miR‐130b	PGC‐1α	^119^
	miR‐143	ERK5	^120^

## 2 PATHOGENESIS OF SARCOPENIA

**FIGURE 1:**
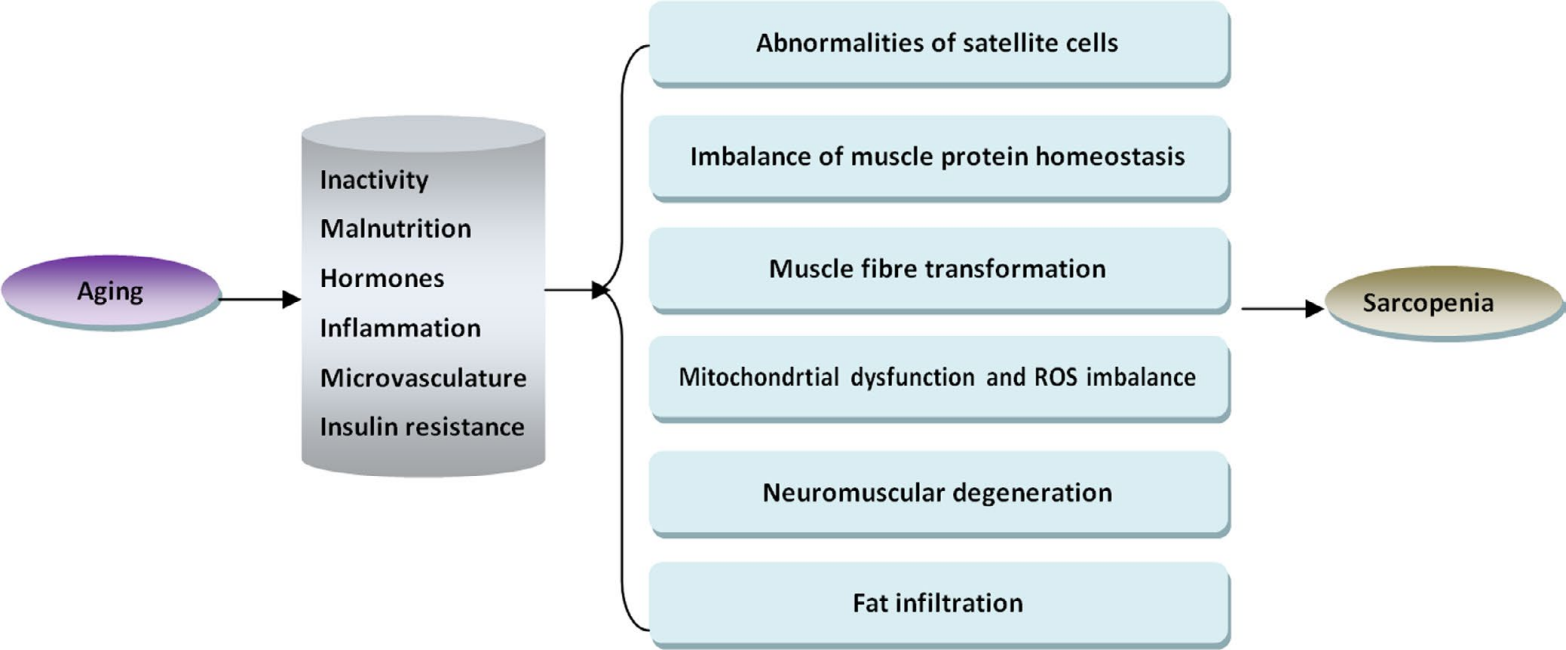
The pathogenesis of sarcopenia during ageing

Sarcopenia is an age‐related disease that is influenced by both environmental and genetic factors. A variety of risk factors, including hormones, inflammation, inactivity, malnutrition, microvasculature and insulin resistance, have been associated with the development of sarcopenia. These factors interact in skeletal muscle in a complex manner, leading to reduced expression of skeletal muscle growth factors and excessive oxidative damage, together with enhanced activity of the ubiquitin‐proteasome system (UPS) and autophagy. These mechanisms result in loss of muscle protein homeostasis, mitochondrial dysfunction and reduced numbers and function of satellite cells, leading eventually to skeletal muscle wasting and dysfunction. Additionally, loss of alpha motor neurons (α‐MNs), alterations in neuromuscular junctions (NMJs) or other factors can contribute to atrophy of muscle fibres, especially type II fibres, and result in a transition of type II muscle fibres to type I muscle fibres. This is accompanied by intramuscular and intermuscular fat infiltration, which leads to abnormal skeletal muscle structure and, eventually, develops into sarcopenia^8^ (Figure 1).

### 2.1 Abnormalities of satellite cells and regenerative capacity

A reduction of approximately 65% in the number of satellite cells, which are also known as adult muscle‐specific stem cells,^9^ and abnormal characteristics of these cells is observed in skeletal muscle of older individuals. This leads to impaired muscle regeneration, reduced antioxidant capacity, increased DNA damage and changes in gene expression.^10^ The process of muscle repair/regeneration is mainly mediated by the activation of satellite cells, which causes the cells to enter the cell cycle, thereby leading to proliferation of myogenic precursor cells, which eventually differentiate and fuse with existing muscle fibres or form new muscle fibres.^11^, ^12^ Paired box‐protein‐7 (PAX7), paired box‐protein‐3 (PAX3) and myogenesis regulator factors (MRFs) constitute key regulatory networks in this process. PAX7, which is the most important marker gene of satellite cells, determines the proliferation and differentiation of satellite cells by regulating genes such as myogenic differentiation (MYOD) and myogenic factor 5 (MYF5), which play important roles in repair following muscle injury.^11^, ^13^, ^14^ Quiescent satellite cells begin proliferation mainly by expression of PAX7 rather than MYOD, MYF5 or other MRFs. Self‐renewal or differentiation of proliferating satellite cells is achieved by maintaining expression of PAX7 and inducing expression of MYOD. By inducing expression of myogenin and down‐regulating expression of PAX7, some of these satellite cells are committed to differentiation. Other satellite cells commit to self‐renewal by inhibiting expression of MYOD. These cells return to the quiescent state to complement the existing quiescent satellite pool, thus ensuring that sufficient numbers of quiescent satellite cells continue to be activated to facilitate subsequent rounds of muscle repair. The intricate transcriptional mechanisms that regulate the quiescence and activation of satellite cells involve about 500 functionally diverse expressed genes. The majority of these are controlled transcriptionally and post‐transcriptionally,^15^ suggesting that changes in satellite cells are key to the development of sarcopenia. It should, however, be noted that some studies have shown that satellite cells may be indispensable in muscle hypertrophy ^16^ and other researchers even believe that satellite cells play a negligible role in sarcopenia.^17^

### 2.2 Imbalance of muscle protein homeostasis

**FIGURE 2:**
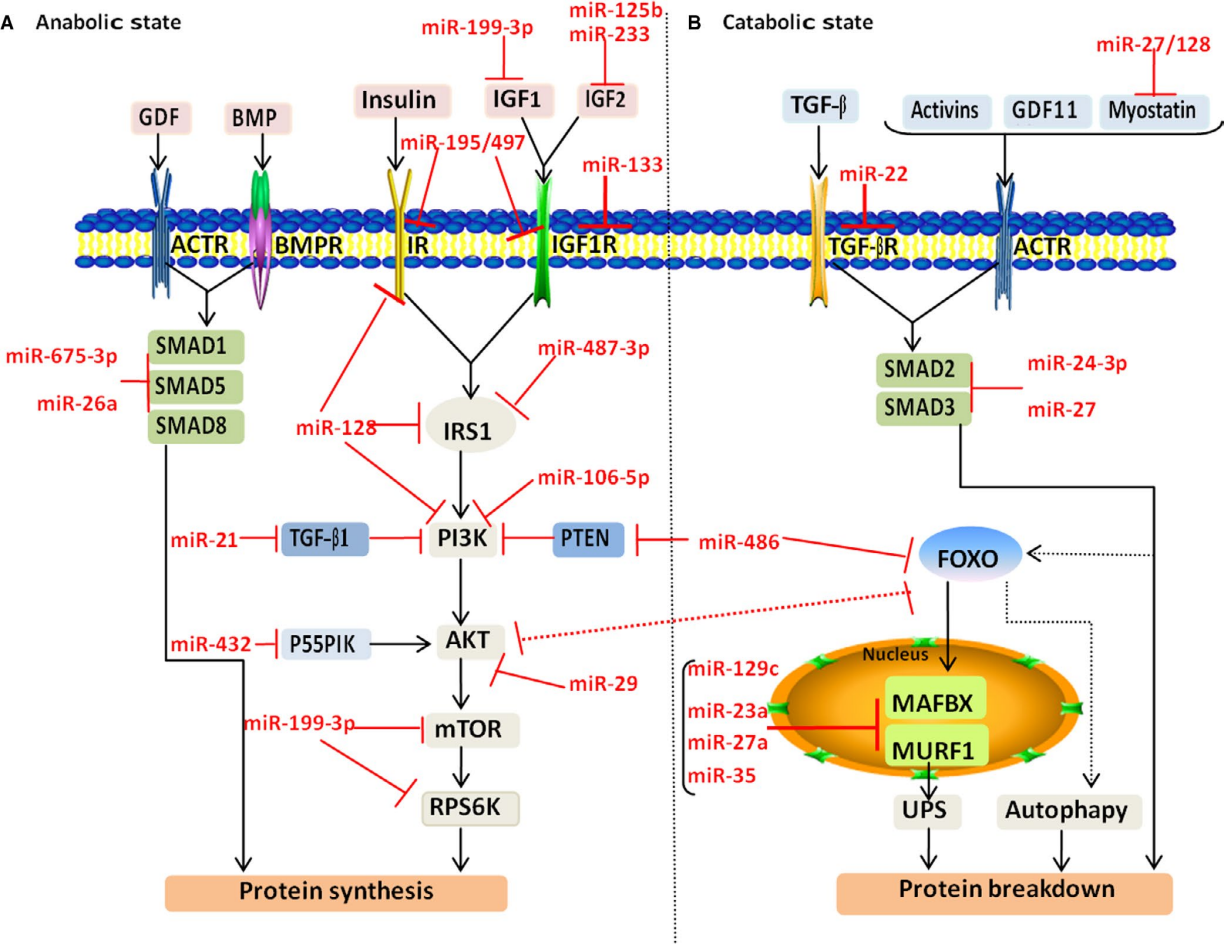
MicroRNA involvement in the homeostasis of muscle protein

Imbalance in protein synthesis and degradation is a major characteristic of muscle wasting in the elderly. It is indisputable that the most important signalling pathway for muscle protein synthesis is the phosphoinositide 3‐kinase/serine‐threonine protein kinase/mammalian target of rapamycin (PI3K/AKT/mTOR) signalling pathway.^18^ Simultaneously, this pathway can indirectly promote protein synthesis by suppressing glycogen synthase‐3 (GSK3) and forkhead box protein O (FOXO),^19^ and by inhibiting the activation of Smad2 by myostatin.^20^ Another pathway involved in protein synthesis is the transforming growth factor‐β (TGF‐β)/myostatin/bone morphogenetic protein (BMP) signalling pathway. Activins and several growth and differentiation factors (GDFs), including myostatin, GDF11 and TGF‐β proteins, depend on Smad2 and Smad3 to regulate target genes and result in protein degradation. In contrast, other GDF ligands or BMP stimulate the activity of Smad1, Smad5 and Smad8 and lead to protein synthesis.^8^ In addition to reduction of anabolic signals, UPS and autophagy are two main protein degradation pathways and play significant roles in sarcopenia.^21^ The UPS induces protein degradation by stimulating expression of muscle atrophy F‐box (MAFBX), and muscle ring finger 1 (MURF1) has been confirmed in some sarcopenia models.^22^, ^23^ The expression of MAFBX and MURF1 is mainly regulated by the transcription factors, nuclear factor‐κB (NF‐κB) and FOXO family members, which are induced by inflammatory factors or hormones.^24^ Another important mechanism for muscle wasting is autophagy, which leads to degradation of mono‐ubiquitinated proteins and is mainly induced by FOXO transcription factors and the AMP‐dependent protein kinase (AMPK) signalling pathway^21^ (Figure 2).

### 2.3 Muscle fibre transformation

During ageing, muscle fibres, especially type II fibres, atrophy and the resulting transition from type II muscle fibres to type I muscle fibres are a prominent feature of sarcopenia.^8^ Adult muscles are composed of different types of muscle fibres and have a wide range of contractile properties.^25^ Different types of muscle fibres express different isoforms of myosin heavy chain (MyHC), including MyHC I, MyHC IIa, MyHC IIx and MyHC IIb, which have distinct oxidative capacities and ATPase activities and which determine the contractility of muscle fibres.^25^ The type of muscle fibres is mainly influenced by loss of α‐MNs, a decrease in motor units, changes in NMJs and denervation of muscle fibres.^8^ Klitgaard *et al* ^26^ showed that coexpression of different isoforms of MyHC in a single muscle fibre increases with age, indicating a continuous change in the type of muscle fibre.^26^ There is a general consensus that muscles in elderly individuals contain more slow muscle fibres,^27^, ^28^ which reduces contractility ability. Some studies, however, have shown no alteration in the type of muscle fibres with ageing,^29^ and some have even reported an increase in the proportion of fast fibres.^30^

### 2.4 Mitochondrial dysfunction and ROS imbalance

The elderly often suffer from mitochondrial dysfunction and overproduction of reactive oxygen species (ROS), which are important causes of sarcopenia.^31^, ^32^, ^33^ Mitochondria are the primary regulators of energy metabolism in the cell and supply 90% of the energy of the cell via oxidative phosphorylation. Mitochondria thus play a central role in cellular homeostasis.^34^ The senescence‐associated secretory phenotype (SASP) of senescent cells, which is characterized by a significant decline in mitochondrial function, an imbalance of metabolism and disrupted homeostasis, leads to ‘signalling noise’ and muscle wasting.^35^ Mitochondrial dysfunction is also closely associated with redox imbalance and overproduction of ROS, which is a product of normal cellular metabolism. ROS are involved in a variety of cellular signal transduction processes and can be accumulated by activated aged satellite cells or proliferating aged myoblasts. The imbalance in ROS, caused by either excess ROS production or defective ROS removal, leads to oxidative stress or damage and results in muscle wasting. An imbalance of ROS may activate aberrant p38 mitogen‐activated protein kinase (MAPK) signalling,^36^, ^37^ deregulate the janus kinase/signal transducer and activator of transcription (JAK‐STAT) signalling pathway,^38^ deregulate expression of the cell proliferation inhibitor, p16INK4a,^39^ and be associated with defective autophagy ^40^ in aged satellite cells. An imbalance in ROS may thus be responsible for age‐related changes in proliferation and differentiation properties. ROS can also accelerate degeneration of skeletal muscle and lead to sarcopenia by activating the UPS and muscle proteases.^41^, ^42^ Excessive accumulation of ROS can damage the structure and function of mitochondria and induce myocyte apoptosis, which leads to dysfunction of the mitochondrial oxidative respiratory chain and increases the production of ROS, eventually forming a vicious cycle.^43^

### 2.5 Neuromuscular degeneration

Normal function of motor neurons is essential for the survival of muscle fibres, and loss of α‐MNs is a key factor in the pathogenesis of sarcopenia. The number of α‐MNs was found to be reduced by up to 50% after the age of 70, which significantly affected the function of muscle.^44^ In the elderly, significant loss of MNs and motor units directly leads to decreased muscle coordination and muscle strength.^44^ Deterioration of neuromuscular communication is another significant contributor to sarcopenia in the elderly. Although it is difficult to determine the roles of α‐MNs and NMJs in regulating skeletal muscle homeostasis during human ageing, recent research using rodent models has highlighted the importance of these factors.^45^, ^46^, ^47^, ^48^ A time‐course analysis in ageing mice clearly showed that, with increasing age, the morphology of the NMJs underwent gradual deterioration, leading to instability of the NMJs.^45^ Bütikofer *et al* ^46^ used mice that overexpress neurotrypsin to destabilize NMJs in skeletal muscle and found that the stability of NMJs plays a key role in regulating muscle fibre mass and function. Regrettably, they did not find that neurotrypsin mediates sarcopenia. Hettwer *et al* ^47^ discovered that administration of agrin fragments maintained body mass during early post‐natal ageing and improved age‐related decrements in muscle function. By accelerating muscle ageing induced by oxidative damage in mice, Sakelariou *et al* ^48^ demonstrated the importance of MNs in maintaining muscle homeostasis. In summary, both reduced quantity and quality of MNs and changes in NMJ morphology play key roles in the formation of an imbalance of muscle homeostasis during ageing. Since nerves affect muscles in many ways, their contribution must not be overlooked when studying age‐related changes in skeletal muscle.

### 2.6 Fat infiltration

Ageing is often accompanied by increased body adiposity and this increase in fat mass is associated with deposition of ectopic fat in tissues, including muscle.^49^ This age‐related fat accumulation seems to have synergistic effects with sarcopenia. The ageing process should be considered as a physiological degradation process, which can be accelerated by concomitant lipotoxic insults.^49^ Several studies have shown that the increase in ectopic fat in muscles of the elderly can lead to reduced strength and performance, which are independently associated with metabolic abnormalities, such as insulin resistance.^50^, ^51^ Several studies have found lipid redistribution and increased lipid deposition in muscle of rats fed a high‐fat diet. A strong negative correlation was shown between myosteatosis and muscle volume, suggesting that ‘lipotoxicity’ may lead to the accumulation of lipids in muscle cells, reduce the rate of protein synthesis and accelerate the development of sarcopenia.^51^, ^52^ Increased catabolism of muscle protein was also seen in rats fed a high‐fat diet, which may be related to increased levels of free fatty acids and decreased levels of adiponectin in plasma.^53^ Fat accumulation in muscle can also affect the function of mitochondria, leading to reduced capacity for oxidation of fatty acids, which has a negative impact on protein metabolism.^51^ Thus, when homeostatic control of adipose tissue is lost, infiltration of fat into skeletal muscle may have a detrimental effect on muscle function and the balance between protein synthesis and degradation.

## 3 MIRNAS AND SARCOPENIA

### 3.1 miRNAs in the regulation of satellite cells and regenerative capacity

miRNAs play key roles in regulating satellite cells and inducing myogenesis by their reciprocal regulatory relationship with multiple transcription factors. miRNAs can influence these processes by targeting several myogenic transcription factors,^54^ and additionally, these transcription factors can also sometimes directly control the expression of miRNAs,^55^ suggesting that miRNAs are an important integral part of the regulatory network in satellite cell‐mediated myogenesis. miRNAs that are specifically, or highly, expressed in skeletal muscle tissue are called myomiRs and include miR‐1, miR‐133a, miR‐133b, miR‐206, miR‐208b, miR‐486 and miR‐499.^56^ These may play an important and dynamic role in myogenesis, not only during embryonic development but also in the adult by maintaining the quiescence and activation of satellite cells.^56^, ^57^, ^58^ miR‐1 and miR‐206 belong to the same family, and during differentiation of muscle satellite cells in mice, miR‐1/206 expression is inversely correlated with expression of Pax7 protein. It has been confirmed that miR‐1/206/486 can inhibit proliferation of satellite cells by targeting the *Pax7* gene and promoting differentiation of myoblasts.^59^, ^60^ Local injection of miR‐1, miR‐133 and miR‐206 has been shown to accelerate muscle regeneration in injured rats, which may be related to increased expression of MRFs, suggesting that miRNAs might be used as a therapeutic strategy against muscle damage.^61^ In mice, miR‐27 can directly target Pax3 to inhibit proliferation and migration and promote differentiation of satellite cells.^62^ It has also been shown that miR‐27 can promote proliferation of satellite cells and muscle fibre hypertrophy by down‐regulating myocyte enhancer factor 2C (Mef2C).^63^ In cytoplasm, miR‐31 and Myf5 mRNA can associate with proteins to form messenger ribonucleoprotein granules, thereby inhibiting post‐transcriptional translation of Myf5 and keeping skeletal muscle satellite cells quiescent.^64^ Additionally, miR‐195 and miR‐497 target *Cdc25* and *Ccnd* genes and miR‐489 targets *DEK* genes to maintain the skeletal muscle satellite cells in a quiescent state.^65^, ^66^ In another study, Let‐7b/e were up‐regulated during ageing of skeletal muscle, possibly affecting the expression of Pax7 through repression of cell cycle regulators, and impeding satellite cell self‐renewal.^67^

### 3.2 miRNAs in muscle protein homeostasis

The PI3K/AKT/mTOR and TGF‐β/myostatin/BMP pathways increase protein synthesis and are vital for myogenic differentiation. Conversely, the UPS and autophagy/lysosome systems activate protein degradation and are detrimental to myogenic differentiation. It has been reported that some miRNAs are involved in regulating several targets of the PI3K/AKT/mTOR pathway in muscle development. For example, miR‐125b and miR‐223, acting through their target insulin‐like growth factor (IGF)‐2, and miR‐199‐3p, acting through IGF‐1, regulate the PI3K/AKT/mTOR pathway and blunt rates of protein synthesis.^68^, ^69^, ^70^ miR‐133, miR‐195 and miR‐497 can target IGF‐1 receptor (IGF‐1R) during skeletal myogenesis.^71^, ^72^ miR‐487b‐3p inhibits protein synthesis through its target insulin receptor substrate 1 (IRS1).^73^ miR‐128 participates in the PI3K/AKT/mTOR signalling pathway by targeting IR, IRS1 and phosphoinositide‐3‐kinase regulatory subunit alpha (PIK3R1).^74^, ^75^ Phosphatidylinositol 3‐kinase regulatory subunit (P55PIK) and PI3K proteins, which are encoded by *PIK3R1*, are targeted by miR‐432 and miR‐106a‐5p, respectively.^76^, ^77^ In addition to targeting IGF‐1, miR‐199‐3p has also been found to target mTOR and ribosomal protein S6 kinase (RPS6K), two significant factors in the PI3K/AKT/mTOR pathway.^70^ miR‐21 also targets transforming growth factor‐beta‐induced (TGFβI) and suppresses PI3K/AKT signalling, thereby regulating skeletal muscle development.^78^ By directly targeting Akt3, miR‐29 appears to participate in regulating the same pathway, leading to inhibition of protein synthesis.^79^ The TGF‐β/myostatin/BMP pathway is also regulated by several miRNAs. For example, TGF‐βI and Smad1 are targeted by miR‐21 and miR‐26a, respectively,^78^, ^80^ transforming growth factor β1 (TGF‐β1) is targeted by miR‐22 and myostatin is targeted by miR‐128.^75^, ^81^ Additionally, miR‐27 regulates the BMP pathway by targeting myostatin and Smad3, miR‐675‐3p regulates the BMP pathway by targeting Smad1 and Smad5,^82^, ^83^, ^84^ and miR‐24‐3p regulates muscle fibrosis by targeting Smad2.^85^ miRNAs are also involved in the UPS and autophagy. MuRF1 and MAFbx, two E3 ubiquitin ligases that are vital for protein degradation during muscle atrophy, were found to be regulated by miR‐23a, miR‐27a and miR‐351.^86^, ^87^ Overexpression of miR‐29c can increase muscle mass by 40% through inhibition of MuRF1.^88^ miR‐486, which targets FoxO1 and phosphatase and tensin homologue (PTEN), also regulates protein degradation by inactivation of skeletal muscle atrophy signalling.^89^ Although specific targets and molecular mechanisms of these differentially expressed miRNAs remain to be explored, they have been demonstrated to play a vital role in muscle protein homeostasis (Figure 2).

### 3.3 miRNAs in different types of muscle fibre

Muscle fibres often change with age, and the principal difference between the different types of fibre is the composition of MyHC isoforms.^90^ Since these MyHC isoforms are encoded by independent genes located at specific genomic loci in humans, it was believed that the composition of different types of muscle fibre was regulated mainly at the transcriptional level,^90^ although this viewpoint has gradually been challenged by myomiRs. miR‐208a, miR‐208b and miR‐499, three types of myomiR from the same family, are embedded in the muscle‐specific MyHC genes *Myh6*, *Myh7* and *Myh7b*, respectively, and play significant roles in regulating the composition of different types of muscle fibre by regulating myosin isoforms.^91^, ^92^ miR‐208a is specifically expressed in cardiac muscle, whereas two other myomiRs, miR‐208b and miR‐499, are also expressed in type I muscle fibres.^91^ miR‐208b and miR‐499 play vital regulatory roles in controlling transformation of muscle fibres, not only by inhibiting fast myofibre‐specific genes but also by activating slow myofibre‐specific genes.^91^, ^92^ Overexpression of miR‐499 has been shown to completely transform fast muscle fibres into slow muscle fibres in soleus muscle.^91^ On the other hand, double knockout of miR‐499 and miR‐208b in mice led to a significant decrease in type I fibres in soleus muscle, decreased expression of MyHC I at both protein and mRNA levels, and increased expression of MyHC IIB and IIX isoforms. On the basis of these findings, van Rooij *et al* ^91^ proposed that the effect of myomiRs on the identity of muscle fibres is mediated mainly by targeting transcriptional inhibitors of slow muscle fibre genes, including Sp3, Sox6, HP‐1β and Purβ. Interestingly, knockout of Sox6 in the skeletal muscle of mice leads to the fast‐to‐slow conversion of muscle fibres.^93^, ^94^ It is noteworthy that myomiRs are all embedded in introns of various MyHC genes and hence have the same expression level as the host gene. On the other hand, these myomiRs can target transcription factors and, in turn, regulate the expression of the MyHC genes. It seems, therefore, that nature uses an endogenous feedback mechanism between miRNAs and MyHC to fine‐tune muscle fibre types and thus regulate muscle physiology and performance.

### 3.4 miRNAs in mitochondria and ROS

Mitochondrial dysfunction and oxidative damage are key processes underlying the majority of age‐related diseases, including sarcopenia. The role of mitochondria in sarcopenia is very suggestive of their role in energy metabolism. miR‐1 not only inhibits cytoplasmic gene expression by targeting HDAC4 and Hand2 but also promotes the expression of mitochondrial genes by targeting mtCox1, mtNd1, mtCytb, mtCox3 and mtAtp8.^95^ This suggests that some miRNAs can coordinate the concurrent translation of nuclear‐encoded and mitochondrial‐encoded mitochondrial proteins. miR‐696 negatively affects fatty acid oxidation and mitochondrial function by targeting the transcription factor peroxisome proliferator‐activated receptor γ coactivator 1α (PGC‐1α), a master regulator of mitochondrial biogenesis and ROS removal.^96^ Another study showed that deficiency of miR‐133a in mice led to low levels of Pgc‐1α and nuclear respiratory factor‐1(Nrf1), and lower mitochondrial mass and exercise tolerance.^97^ This phenotype is similar to the sarcopenia phenotype, suggesting that miR‐133a has a significant role in maintaining skeletal muscle mitochondrial dynamics. Overexpression of miR‐23 in amyotrophic lateral sclerosis (ALS) patients also represses the expression of PGC‐1α, resulting in mitochondrial dysfunction.^98^ By regulating B‐cell lymphoma‐2 (Bcl‐2), several ageing‐related mitomiRs (−34a −146a and −181a) may also play direct roles in controlling mitochondrial function by regulating the expression of mitochondrial proteins. The resulting loss of mitochondrial function and integrity would result in inflammation, elevated levels of ROS, apoptosis and age‐related diseases, assuming that some typical alterations of senescence may depend on the deregulation of mitomiRs.^99^, ^100^ miR‐340‐5p and miR‐206 have also been shown to regulate ROS generation in skeletal muscle via nuclear respiratory factor‐1(Nrf2), which is a key factor in regulating redox homeostasis. Whether these miRNAs are involved in sarcopenia remains unknown.^101^, ^102^

### 3.5 miRNAs in neurodegeneration

Changes in MNs and NMJ morphology are important features in the elderly with sarcopenia. The function of skeletal muscle requires innervation by the central nervous system to form the motor units of muscle. Dysfunction or loss of innervation can lead to a variety of muscle disorders, in which miRNAs play an indispensable role. It has been demonstrated that miR‐206, which is mainly expressed in skeletal muscle, is a key regulator of signals between MNs and NMJs.^13^ In mouse models, miR‐206 delayed the progression of experimental ALS and promoted regeneration of neuromuscular synapses.^13^ Gene ablation of miR‐206, on the other hand, accelerated disease progression, delayed NMJ regeneration and reinnervation, and subsequently accelerated skeletal muscle atrophy.^103^, ^104^ Additionally, by targeting tumour suppressor p53, miR‐375 also plays a protective role in the development of MNs.^15^ In mice with spinal muscular atrophy, miR‐146a is up‐regulated and overexpression of miR‐146a tends to induce loss of MNs, whereas inhibiting miR‐146a prevents loss of MNs ^16^. miR‐23a was also shown to significantly reduce pathology in mice with spinal muscular atrophy by increasing the size of motor neurons, decreasing pathology associated with NMJs, increasing the area of muscle fibres and prolonging survival ^17^. Overexpression of miR‐234 has been found to endow resistance to the acetylcholinesterase inhibitor aldicarb, suggesting that miR‐234 can control neuropeptide levels and lead to NMJ dysfunction ^18^. During the development of drosophila, let‐7 and miR‐125 control the maturation of NMJs by targeting *abrupt* genes ^109^, ^110^. miR‐1 also participates in the regulation of NMJs by a MEF2‐dependent mechanism ^111^. These studies indicate that miRNAs play key roles in regulating the quality and quantity of MNs and NMJs. There have been, however, few studies in models of sarcopenia, and more research is needed to clarify whether miRNAs play roles in regulating neurodegeneration in sarcopenia.

### 3.6 miRNAs in fat infiltration

In sarcopenia, muscle wasting is commonly associated with fat infiltration. During muscle atrophy caused by ageing and malnutrition, fibro‐adipogenic progenitors (FAPS) are uncontrollably activated and differentiated into fibroblasts and adipocytes, leading to infiltration of adipose tissue and fibrotic scars, which limits proper muscle regeneration and repair.^112^, ^113^ MyomiRs (miR‐1‐2, miR‐133 and miR‐206) have been shown to regulate the FAP functional phenotype in malnourished mice by regulating the ATP‐dependent BRG1/BRM associated factor (BAF) subunits, which may contribute to deposition of ectopic adipose tissue.^114^ Additionally, miR‐133 and miR‐499 regulate brown adipocyte differentiation by directly targeting PR domain containing 16 (PRDM16), the major transcription factor for brown adipogenesis.^115^, ^116^ By inhibiting the differentiation of platelet‐derived growth factor receptor α^+^ (PDGFRα^+^) progenitor cells into adipocytes, overexpression of miR‐23a was shown to reduce lipid accumulation in skeletal muscle.^117^, ^118^ miR‐130b, secreted from adipocytes, was able to target muscle cells and reduce expression of PGC‐1α, which plays an important role in lipid oxidation in muscle.^119^ Additionally, miR‐143 promoted synthesis of triglycerides, both in humans and rodents, which may contribute to infiltration of adipocytes.^120^ These data suggest that miRNAs may participate in accumulation of fat during sarcopenia.

## 4 PROSPECTS OF MIRNA THERAPY FOR SARCOPENIA

There is currently no particularly effective treatment strategy for sarcopenia, and reasonable exercise and a proper diet are typically recommended as safe, validated, low cost, non‐pharmacological interventions. Assistive treatments, such as hydrotherapy, vibration therapy and functional electrical stimulation, may also be used as supplementary procedures. Pharmacological treatments, especially hormone preparations, produce marked benefits but the side effects are also very prominent. MiRNAs are endogenous and multifunctional small molecules with limited potential for immunogenicity to the human genome, and they have numerous activities in various biological processes and promise to become a new therapy for diseases. Investigation of miRNA therapy for challenging diseases remains an area of great interest.

Patisiran has received global approval in 2018 for the treatment of transthyretin‐mediated amyloidosis, founding the first RNA interference (RNAi)‐based therapeutic worldwide.^121^ The discovery of RNAi technology greatly broadens the source and development direction of human drugs. This is a more specific and effective treatment strategy. In addition, RNAi technology has other advantages, such as short pre‐clinical development cycle and abundant candidate targets. RNA‐based therapeutics are expected to become a rapidly growing market and gain more and more commercial interests. Unfortunately, there are no miRNA‐based drugs have been used to the clinic yet and only a few drugs have entered phase I or phase II clinical trials (Table 2). Miravirsen, an antisense to human miRNA‐122, has reached phase II clinical trials for hepatitis C virus infection and appears to be well tolerated and to have no dose‐limiting toxicity.^122^ Unfortunately, MRX34, a liposomal miR‐34 mimetic for the treatment of liver cancer, phase I clinical trials were halted for immune‐related serious adverse events.^123^ In addition, many other miRNA‐based therapies are in the pre‐clinical stage. There are many limitations and difficulties in miRNA‐based therapies that lead to their current lack of success. Identification of miRNA targets of interest remains an important challenge, and the heterogeneity of miRNA expression continues to hinder the development of miRNA drugs.^124^ Ensuring target specificity and limiting unnecessary off target effects and toxicity are also significant hurdles for miRNA drugs.^125^ Other issues that need to be urgently addressed include the development of appropriate delivery systems to provide safety and stability, understanding the long‐term effects of regulating miRNA activity, establishing the safety and efficacy of miRNA‐based therapeutics and modelling the pharmacodynamics and pharmacokinetics of these miRNA drugs.

**TABLE 2:** miRNA‐based treatment in clinical trials

Name	Therapeutic agent	Delivery system	Target disease	Stage	National clinical trials number
MesomiR‐1	miR‐16 mimic	EnGeneIC Dream Vector	Malignant pleural mesothelioma non‐small cell lung cancer	Phase I	NCT02369198
MRG‐201	miR‐29 mimic	Cholesterol‐conjugated miRNA duplex	Keloid	Phase II	NCT03601052
MRG‐201	miR‐29 mimic	Cholesterol‐conjugated miRNA duplex	Fibrosis	Phase I	NCT02603224
Miravirsen	Anti‐miR‐122	LNA antagomir	Hepatitis C	Phase II	NCT02452814 NCT02508090
RG‐012	Anti‐miR‐21	NA	Hereditary nephritis	Phase II	NCT02855268
MRG‐106	Anti‐miR‐155	LNA‐modified antisense inhibitor	CTCL; MF	Phase II	NCT03713320 NCT03837457
MRG‐106	Anti‐miR‐155	LNA‐modified antisense inhibitor	CLL; DLBCL; ATLL	Phase I	NCT02580552
MRG‐110	Anti‐miR‐92	LNA antagomir	Wounds	Phase I	NCT03603431

With ageing, deregulation of miRNAs in muscle is closely associated with sarcopenia, suggesting that miRNAs may be a viable therapeutic target for sarcopenia. Compared with studies of miRNA in diseases such as cancer and heart disease, the study of miRNA function in skeletal muscle, especially in sarcopenia, is still in its infancy and the roles and specific mechanisms of miRNAs in sarcopenia need to be further elucidated. Efficient delivery systems and stable therapeutic molecules will be key to the development of miRNA‐based therapy for muscle diseases, including sarcopenia. Increasing numbers of clinical trials of miRNA‐based therapies are under way, and these should provide proof of principle for the use of miRNA to fight refractory diseases. The development of miRNA‐based therapy for sarcopenia will inevitably face challenges but, once these obstacles are overcome, miRNA‐based therapy may provide a welcome strategy for the treatment of sarcopenia.

## 5 CONCLUSIONS

Loss of skeletal muscle is a major problem associated with ageing. The decline in muscle quantity and quality leads to decreased quality of life and increased mortality. Many studies have sought to identify the intrinsic and external factors that cause muscle ageing and to discover targeted interventions for age‐related muscle atrophy. miRNAs in mammalian genomes are essential for the development and function of life, and a large number of miRNAs are important regulators. Their expression profiles change with age, functionally contributing to ageing‐related loss of muscle quantity and quality. With continuous updating of technology to detect miRNAs, the understanding of the roles of miRNAs as a whole will become more comprehensive. This review article describes how, by negatively regulating the expression of target genes, miRNAs modulate the processes involved in sarcopenia and also discusses the prospect of miRNAs as a treatment for sarcopenia. In summary, miRNAs are promising small molecule therapeutics and it is hoped that, through both large scale validation research and carefully designed functional research using in vitro and in vivo systems, miRNAs will, in the future, become an effective means of treating refractory diseases.

## CONFLICT OF INTEREST

The authors declare that there are no conflicts of interest.

## AUTHOR CONTRIBUTION

All authors have contributed to the writing of the manuscript.
